# Association between depressive symptoms and balance disorders in older adults living in 12 high Andean communities

**DOI:** 10.1371/journal.pone.0353158

**Published:** 2026-07-08

**Authors:** Maite T. Alanya-Pineda, Ivonne A. Bravo-Alcántara, Ana L. Alcantara-Diaz, Leslie Salazar-Talla, Diego Urrunaga-Pastor, Fernando M. Runzer-Colmenares, José F. Parodi

**Affiliations:** 1 Facultad de Ciencias de la Salud, Universidad Científica del Sur, Carrera de Medicina Humana, Lima, Peru; 2 CHANGE Research Working Group, Carrera de Medicina Humana, Universidad Científica del Sur, Lima, Peru; 3 Facultad de Medicina Humana, Universidad de San Martin de Porres, Centro de investigación del envejecimiento (CIEN), Lima, Peru; Universidad de Las Americas, Quito-Ecuador, ECUADOR

## Abstract

**Background:**

The prevalence of depressive symptoms and balance disorders may be higher in high-altitude Andean regions due to chronic hypoxia, neurochemical alterations, and limited access to health services. Since both conditions can coexist and contribute to functional decline and falls, their association is relevant. We aimed to estimate the association between depressive symptoms and balance disorders in older adults living in 12 high Andean communities.

**Methods:**

We carried out a secondary analysis of data from a cross-sectional analytical study in older adults residing in 12 Peruvian high Andean communities during the period 2013–2020. The exposure variable was depressive symptoms (defined as a score greater than or equal to two on the five-item geriatric depression scale), while the outcome variable was balance disorders (defined by a functional reach test less than or equal to 20.32 cm). We constructed generalized linear models from Poisson family with link log and robust variances. We estimated crude (cPR) and adjusted (aPR) prevalence ratios with their respective 95% confidence intervals (95%CI).

**Results:**

We analyzed 417 older adults; 61.1% (n = 255) were women, with a mean age of 73.2 ± 6.9 years. Additionally, 52.8% (n = 220) presented depressive symptoms, while 48.9% (n = 204) presented balance disorders. In the adjusted regression model, depressive symptoms were associated with a higher prevalence of balance disorders in older adults (aPR = 1.66; 95%CI: 1.28–2.15; p < 0.001).

**Conclusions:**

Depressive symptoms were associated with a higher prevalence of balance disorders in older adults residing in the 12 high Andean communities. Future epidemiological studies with a larger sample size are needed to evaluate depressive symptoms and balance disorders to develop early screening programs in older adults to improve their quality of life and access to primary health care.

## 1. Introduction

Aging of the population is advancing by leaps and bounds, with an expected global population of older adults of 22% by the year 2050 [1]. As aging progresses the effects of stress on the central nervous system are exacerbated, leading to the development and increase of cognitive and behavioral changes such as those of the depressive syndrome in older adults [2,3]. Likewise, since depression is a multifactorial disorder [4], the presentation and development of which involve a series of biological [5] and sociodemographic [6] factors, it can be triggered by multiple pathways. The estimated global prevalence of depression in older adults is 7% worldwide [1]. In Latin American countries, the highest prevalence of depression was found in Mexico (26%), Peru (16%) and Colombia (11.5%) [7]. Despite the high prevalence of depressive symptoms, a large proportion of older adults are not diagnosed and treated in a timely manner due to various factors such as living in middle and low-income countries, widowhood or residing in rural areas [8–10]. Factors associated with depression have been described in the literature, including female biological sex, living under stress, loneliness, some degree of dependence, functional impairment, frailty and living in a rural area, with the latter also being a contributing factor to the development of balance disorders, likely due to limited access to healthcare, irregular terrain, and reduced physical rehabilitation services [11,12].

Depressive symptoms can influence the presence of balance disorders [13], which is a predisposing factor to the incidence of falls [14]. Balance disorders were caused by physiological changes associated with structural and functional impairment, compromising postural control and gait [15]. The study in older adults by Dutta *et al.* reported that as depression increases, balance decreases [13] since both share physiological changes in the nervous, sensory, and musculoskeletal systems, producing balance disorders [16]. Likewise, residing in high-altitude areas can cause alterations in serotonin metabolism, which has been proposed as a cause of depression [6,17,18]. Residents of high altitude can present dizziness, instability, and vertigo, which are mediated by hypoxia and favor the development of balance disorders [19,20]. Therefore, older adults living at high altitudes can present a higher prevalence of depressive symptoms and balance disorders, which can be evaluated by means of the functional reach test, which is easy to use and administer in geriatric populations [21].

Previous studies in Turkey and India found an association between depression and balance disorders; however, none of these studies included older adults living at high altitudes [11,13,22,23]. In Peru, no study has evaluated this association; however, the prevalence of depression in older adults living at high-altitude is 26.9% [24], while the prevalence of balance disorders in the general population living at high-altitude is 27.3% to 28.9% [25], which are alarming figures. It is relevant to study this association, since it will allow establishing preventive interventions for populations at high risk of depression, such as older adults [26]. We hypothesize that there may be an even greater probability of balance disorders and depression among older adults residing in high-altitude areas. For this reason, in the present study we aimed to evaluate whether there is an association between depressive symptoms and balance disorders in the older adult population residing in 12 high Andean communities.

## 2. Methods

### 2.1. Study design, population, and sample

We conducted a secondary analysis of data from a cross-sectional study [27]. The primary study included 453 older adults recruited by census sampling, representing 95% of persons aged 60 years or older residing in 12 high Andean communities, evaluated during 2013–2020. Previous studies have been conducted using this database [27,28]. Residents of the following high Andean communities located above 1,500 meters above sea level (masl) were recruited from the departments of: (a) Amazonas (La Jalca and Leimebamba), (b) Ancash (Atipayán, Llupa, Macashca and San Pedro de Chaná), (c) Ayacucho (Ayahuanco), (d) Huancavelica (Vilca), (e) Huancayo (Chacapampa), (f) Huánuco (Pampamarca), (g) Lima (Viñac), and (h) Puno (Paucarcolla) [28]. For this secondary analysis, we excluded participants with missing data on the variables of interest, resulting in a final sample of 417 older adults. For the calculation of statistical power, the study by Launay *et al.* [29], was used, in which depressive symptoms and the prevalence of falls (a variable associated with balance disorders) were compared. The proportion of falls in the group with depressive symptoms was 29.6%, while the proportion of falls in the group without depressive symptoms was 14.8%, considering a 95% confidence level, providing a statistical power of 99.9% for a sample of 417 older adults.

### 2.2. Procedure

The data were obtained by means of surveys and anthropometric measurements taken by trained medical personnel, including general practitioners, geriatricians, and medical students, who visited the inhabitants in their homes up to three times to invite them to voluntarily participate in the study, after reading the informed consent form. A form was filled out in which sociodemographic data, medical and personal history (tobacco and alcohol consumption, comorbidities, and body mass index (BMI), neuropsychiatric evaluation (depressive symptoms and cognitive impairment), and functional evaluation (Edmonton test, and functional reach test) were collected.

In the primary study, we excluded older adults with severe cognitive impairment (greater than or equal to eight points) assessed by the Pfeiffer questionnaire, as well as individuals unable to understand or communicate in Spanish or for whom a translator was not available, those with hearing or visual impairments that hindered the application of the survey, persons presenting any physical disability that did not allow the execution of physical performance tests, and finally, individuals without the variables of interest for the elaboration of the present study.

### 2.3. Variables

#### 2.3.1. Outcome variable: Balance disorders.

The evaluation of balance disorders was carried out by means of the functional reach test, a validated clinical measure of dynamic balance. For the execution of the test, the participant stood upright with feet shoulder-width apart alongside a wall-mounted measuring scale. The dominant arm was raised to 90° of shoulder flexion with the elbow extended and fist closed. The participant was then instructed to reach maximally forward without stepping or losing balance. The displacement of the third metacarpal head from the starting to the maximum forward position was recorded in centimeters (cm), considering the best of two attempts. A balance disorder was defined as achieving a functional reach value less than or equal to 20.32 cm (8 inches), a threshold validated for fall risk identification in community-dwelling older adults [30–33].

#### 2.3.2. Exposure variable: Depressive symptoms.

Depressive symptoms were assessed using the five-item Geriatric Depression Scale (GDS-5), a brief screening tool derived from the original 30-item GDS. The GDS-5 consists of five yes/no questions, and a score greater than or equal to 2 was considered a positive screen for depressive symptoms [34,35]. The instrument has been validated in Spanish-speaking populations with an adequate diagnostic performance (sensitivity of 78–97% and specificity of 69–96% against structured clinical interviews in geriatric populations) [36,37].

#### 2.3.3. Other variables.

##### 2.3.3.1. Sociodemographic characteristics:

The following sociodemographic characteristics were collected by self-reporting: sex (female, male), age (less than or equal to 70 years, 71–80 years and over 80 years), marital status (single, married and widowed/divorced), educational level (no education/incomplete primary, complete primary and secondary), living alone (no, yes), having a job (no, yes), altitude of residence (masl) and verified by reviewing the national identity card (NIC) of each participant and information provided by the participant’s companion during data collection.

##### 2.3.3.2. Medical and personal history

The following variables were assessed by self-reporting: tobacco consumption (no, yes), alcohol consumption (no, yes), and comorbidities including high blood pressure (HBP) (no, yes), chronic obstructive pulmonary disease (COPD) (no, yes), type 2 diabetes (T2D) (no, yes), low back pain (no, yes), urinary incontinence (using the following question from the Edmonton questionnaire: “Do you have trouble holding your urine when you don’t feel like urinating?” [38]) (no, yes) and polypharmacy (defined as five or more commonly used drugs are prescribed [39]) (no, yes). In addition, a variable was created that included the previously described comorbidities, using the following categories: zero, one, and two or more. BMI was also considered, using the following formula: weight(kg)/[height(m^2^)].

##### 2.3.3.3. Social assessment

Social support was assessed using the following question from the Edmonton questionnaire: “When you need help, do you have someone who meets your needs?” (always, sometimes/never) [38].

##### 2.3.3.4. Neuropsychiatric evaluation

The presence of neurocognitive disorders was assessed using the 10-item Pfeiffer questionnaire, with categorization according to the number of errors obtained in answering each question: zero to two errors (no neurocognitive disorder), three to four errors (mild neurocognitive disorder), and five to seven errors (moderate neurocognitive disorder) [40].

### 2.4. Statistical analysis

Data analysis was performed with the statistical package Stata/SE v17.0 (StataCorp, TX, US). For descriptive analysis, numerical variables were presented using means and standard deviation, as well as medians with their respective interquartile ranges, depending on the normality of the distribution of the variable. On the other hand, relative and absolute frequencies were used to summarize the categorical variables. Similarly, we used the Pearson’s Chi-square test or the Fisher’s exact test for comparison between categorical variables. Likewise, the Student’s-t test or the Mann Whitney U test was used to compare the differences of a numerical variable between groups according to the presence of depressive symptoms and balance disorders.

Two generalized linear models (crude and adjusted) of the Poisson family with logarithmic link function with robust variance were constructed with the aim of assessing the association between depressive symptoms and balance disorders in older adults. In this way, we estimated crude (cPR) and adjusted (aPR) prevalence ratios with their respective 95% confidence intervals (95%CI). We pre-specified a minimal sufficient adjustment set under an epidemiological criterion guided by prior literature [4–6,8,41–43]. A variable qualified as a confounder if it was a plausible common cause of both depressive symptoms and balance impairment, preceded both processes, and was not on the causal pathway nor a descendant of either. Accordingly, the adjusted model included sex, age, educational level, comorbidities, BMI, social support, and neurocognitive disorder [4–6,8,41–43]. Covariates were selected a priori, based on prior literature and not by statistical significance in bivariate analysis; bivariate results are presented only to describe the sample. We assessed multicollinearity in the adjusted model using variance inflation factors (VIFs).

### 2.5. Ethical approval

The primary study was evaluated and approved by the Institutional Research Ethics Committee (IREC) of the Naval Medical Center (Memorandum N°49). The study was conducted in accordance with the ethical principles outlined in the Declaration of Helsinki and included obtaining informed consent from all older adults participating in the study. Neither the integrity nor the confidentiality of the data provided was violated. Likewise, the present secondary analysis did not violate participant confidentiality and was evaluated and approved by the IREC of Universidad Científica del Sur (No. 011-DACMH-DAFCS-U. CIENTIFICA-2022).

## 3. Results

### 3.1. Descriptive and bivariate analysis according to the prevalence of balance disorders in the study sample

Data from 417 older adults residing in 12 high Andean communities in Peru were analyzed. A total of 255 (61.1%) women were included, with a mean age of 73.2 ± 6.9 years, residing at a median altitude of 3,400 masl (3,315−3,445), and 220 (52.8%) and 204 (48.9%) of the participants had depressive symptoms and balance disorders, respectively (Table 1). Likewise, the mean functional reach was 19.9 ± 6.5 cm. Table 1 shows the characteristics according to the prevalence of balance disorders in older adults. We found that marital status (p = 0.007), altitude of residence (p = 0.013), tobacco consumption (p = 0.002), alcohol consumption (p < 0.001), polypharmacy (p = 0.001), BMI (p = 0.002), social support (p = 0.016), and neurocognitive disorder (p < 0.001) presented statistically significant differences according to the presence of balance disorders.

**Table 1:** Descriptive and bivariate analysis of the variables of interest according to the prevalence of balance disorders in older adults in 12 high Andean communities of Peru (n = 417).

			Balance disorders		
			Normal	Altered	P value
Variables	n	%	n=213 (51.1%)	n=204 (48.9%)	
Sex					0.291
Female	255	61.1	125 (49.0)	130 (51.0)	
Male	162	38.9	88 (54.3)	74 (45.7)	
Age	73.2 ± 6.9		72.9 ± 6.6	73.4 ± 7.2	0.471
≤70 years	165	39.5	87 (52.7)	78 (47.3)	0.853
71-80	185	44.4	93 (50.3)	92 (49.7)	
>80	67	16.1	33 (49.3)	34 (50.7)	
Marital status					**0.007**
Single	43	10.3	26 (60.5)	17 (39.5)	
Married	250	60.0	112 (44.8)	138 (55.2)	
Widowed/divorced	124	29.7	75 (60.5)	49 (39.5)	
Educational level					0.089
No education/Incomplete elementary school	333	79.8	169 (50.8)	164 (49.2)	
Complete primary school	74	17.8	42 (56.8)	32 (43.2)	
Complete secondary school	10	2.4	2 (20.0)	8 (80.0)	
Living alone					0.059
No	325	77.9	158 (48.6)	167 (51.4)	
Yes	92	22.1	55 (59.8)	37 (40.2)	
Work					0.194
No	197	47.2	94 (47.7)	103 (52.3)	
Yes	220	52.8	119 (54.1)	101 (45.9)	
Altitude	3400 (3315-3445)		3400 (2158-3511)	3400 (3275-3445)	**0.013**
Tobacco consumption					**0.002**
No	368	88.3	198 (53.8)	170 (46.2)	
Yes	49	11.7	15 (30.6)	34 (69.4)	
Alcohol consumption					**<0.001**
No	301	72.2	182 (60.5)	119 (39.5)	
Yes	116	27.8	31 (26.7)	85 (73.3)	
Comorbidities					0.090
0	186	44.6	106 (57.0)	80 (43.0)	
1	155	37.2	73 (47.1)	82 (52.9)	
2 or more	76	18.2	34 (44.7)	42 (55.3)	
HBP	45	10.8	17 (37.8)	28 (62.2)	0.059
COPD	17	4.1	8 (47.1)	9 (52.9)	0.735
T2D	34	8.2	16 (47.1)	18 (52.9)	0.625
Low back pain	78	18.7	35 (44.9)	43 (55.1)	0.224
Urinary incontinence	142	34.1	73 (51.4)	69 (48.6)	0.923
Polypharmacy	13	3.1	1 (7.7)	12 (92.3)	**0.001**
BMI	25.6 ± 3.7		25.1 ± 3.4	26.3 ± 4.0	**0.002**
Social support					**0.016**
Always	174	41.7	101 (58.1)	73 (41.9)	
Sometimes/never	243	58.3	112 (46.1)	131 (53.9)	
Neurocognitive disorder					**<0.001**
No	257	61.6	124 (48.3)	133 (51.7)	
Mild	122	29.3	81 (66.4)	41 (33.6)	
Moderate	38	9.1	8 (21.1)	30 (78.9)	
Depressive symptoms					0.211
No	197	47.2	107 (54.3)	90 (45.7)	
Yes	220	52.8	106 (48.2)	114 (51.8)	

HBP: high blood pressure; COPD: chronic obstructive pulmonary disease; T2D: type 2 diabetes; BMI: body mass index.

### 3.2. Descriptive and bivariate analysis according to depressive symptoms in the study sample

The presence of depressive symptoms was significantly associated with age (p < 0.001), older adults younger than or equal to 70 years of age (p = 0.003), living alone (p = 0.045), tobacco consumption (p < 0.001), polypharmacy (p = 0.019), urinary incontinence (p < 0.001), social support (p < 0.001), and neurocognitive disorder (p < 0.001) (Table 2).

**Table 2:** Descriptive and bivariate analysis of the variables of interest according to the prevalence of depressive symptoms in older adults in 12 high Andean communities of Peru (n = 417).

	Depressive symptoms		
	No	Yes	P value
Variables	n=197 (47.2%)	n=220 (52.8%)	
Balance disorders			0.211
No	107 (50.2)	106 (49.8)	
Yes	90 (44.1)	114 (55.9)	
Sex			0.362
Female	125 (49.0)	130 (51.0)	
Male	72 (44.4)	90 (55.6)	
Age	71.8 ± 6.6	74.4 ± 7.0	**<0.001**
≤70 years	92 (55.8)	73 (44.2)	**0.003**
71-80	84 (45.4)	101 (54.6)	
>80	21 (31.3)	46 (68.7)	
Marital status			0.686
Single	23 (53.5)	20 (46.5)	
Married	116 (46.4)	134 (53.6)	
Widowed/divorced	58 (46.8)	66 (53.2)	
Educational level			0.648
No education/Incomplete elementary school	158 (47.5)	175 (52.5)	
Complete primary school	33 (44.6)	41 (55.4)	
Complete secondary school	6 (60.0)	4 (40.0)	
Living alone			**0.045**
No	162 (49.9)	163 (50.1)	
Yes	35 (38.0)	57 (62.0)	
Work			0.194
No	88 (44.7)	109 (55.3)	
Yes	109 (49.5)	111 (50.5)	
Altitude	3400 (3275-3414)	3413 (3336-3511)	0.157
Tobacco consumption			**<0.001**
No	186 (50.5)	182 (49.5)	
Yes	11 (22.5)	38 (77.5)	
Alcohol consumption			0.073
No	134 (44.5)	167 (55.5)	
Yes	63 (54.3)	53 (45.7)	
Comorbidities			0.062
0	77 (41.4)	109 (58.6)	
1	84 (54.2)	71 (45.8)	
2 or more	36 (47.4)	40 (42.6)	
HBP	17 (37.8)	28 (62.2)	0.178
COPD	7 (41.2)	10 (58.8)	0.609
T2D	17 (50.0)	17 (50.0)	0.737
Low back pain	33 (42.3)	45 (57.7)	0.333
Urinary incontinence	86 (60.6)	56 (39.4)	**<0.001**
Polypharmacy	2 (15.4)	11 (84.6)	**0.019**
BMI	25.9 ± 4.1	25.4 ± 3.4	0.198
Social support			**<0.001**
Always	119 (68.4)	55 (31.6)	
Sometimes/never	78 (32.1)	165 (67.9)	
Neurocognitive disorder			**<0.001**
No	170 (66.2)	87 (33.8)	
Mild	23 (18.9)	99 (81.1)	
Moderate	4 (10.5)	34 (89.5)	

HBP: high blood pressure; COPD: chronic obstructive pulmonary disease; T2D: type 2 diabetes; BMI: body mass index.

### 3.3. Crude and adjusted prevalence ratios to estimate the association between depressive symptoms and balance disorders in the study sample

We found that the presence of depressive symptoms was associated with a higher prevalence of balance disorders (aPR = 1.66; 95%CI: 1.28–2.15; p < 0.001) after adjusting for sex, age, educational level, comorbidities, BMI, social support, and neurocognitive disorder (Table 3).

**Table 3:** Crude and adjusted regression models to estimate the association between depressive symptoms and balance disorders in older adults in 12 high Andean communities of Peru.

	Crude			Adjusted		
Depressive symptoms	cPR	95%CI	P value	aPR*	95%CI	P value
No	Reference	–	–	Reference	–	–
Yes	1.13	0.93-1.38	0.214	1.66	1.28-2.15	**<0.001**

* Adjusted for: sex, age, educational level, comorbidities, BMI, social support, and neurocognitive disorder.

cPR: crude prevalence ratio; aPR: adjusted prevalence ratio; CI: confidence interval.

## 4. Discussion

### 4.1. Main findings

We found that five out of ten older adults had depressive symptoms or balance disorders. The presence of depressive symptomatology was associated with a higher prevalence of balance disorders in older adults living in 12 Peruvian high Andean communities. The difference between the crude and adjusted prevalence ratios reflects negative confounding. In the crude analysis, the reference group of older adults without depressive symptoms was disproportionately burdened by neurocognitive disorder and lack of social support, two factors independently associated with worse balance, affecting the crude prevalence ratio and attenuating the true association. Once these imbalances were accounted for in the adjusted model, the association between depressive symptoms and balance disorders appeared with its actual magnitude. These findings support the development of targeted interventions for older adults residing at high altitude. Non-pharmacological approaches with evidence in older adult populations include balance and resistance training programs [44] and cognitive-behavioral therapy. Regarding pharmacological interventions, selective serotonin reuptake inhibitors (SSRIs) may improve depressive symptoms; however, long-term antidepressant use has also been associated with balance and gait impairment in older adults [45,46]. Therefore, a personalized, multi-component approach to treatment is recommended.

### 4.2. Comparison with previous studies

We found that depressive symptoms are associated with an increased likelihood of balance disorders in older adults from 12 high Andean communities, which is consistent with findings reported by a previous systematic review [11] and a study in India [23]. However, a study performed in Turkey did not find the present association [22], while a study in India reported that higher depression scores were associated with worse balance performance [13], which is directionally consistent with our findings. It should be noted that no study exploring this association in populations residing at high altitudes were found. However, previous studies have reported the prevalences and averages of depressive symptoms and balance disorders in residents of high-altitude communities. Previous studies in the Andes [47] and Himalayan regions [7,24,47] reported a lower prevalence of depressive symptoms (1.8% to 26.9%) than that found in our study (52.8%) in adults and older adults. These differences might be explained using different depressive symptom assessment instruments (the PHQ-9, the PHQ-2 and the 10-question GDS), collection time and sample size [7,24,47]. On the other hand, previous studies on balance disorders in the Himalayas and Karakoram [19,48] conducted in older adults, reported means between 13.7 cm to 26.5 cm in the functional reach test, similar to the values described in our study.

### 4.3. Interpretation of the findings

Hypobaric hypoxia affects both brain bioenergetics and serotonin metabolism, both of which contribute to the development of depression [6]. Likewise, the presence of motor abnormalities affecting gait, posture and balance are described as part of the pathophysiology of depression, as well as its association with a worse response to treatment [11]. In addition, hypobaric-normobaric hypoxia also influences inspired, arterial and alveolar oxygen partial pressure, central nervous system integration and sensorimotor function, all of which are involved in the control of standing balance [12]. Therefore, residents at high altitudes may have a higher prevalence of depressive symptoms and balance disorders as found in the present study. In addition, it has been reported that individuals presenting both conditions present greater executive dysfunction, as well as a greater risk of falls [49], which is important in the older adult population, since falls represent one of the main causes of morbidity and mortality [50]. Moreover, executive dysfunction may suggest that the older adult is in a mild stage of Alzheimer’s disease [51,52]. Another important aspect of evaluation is vascular diseases, because the burden of lesions in the white matter can cause simultaneous changes in gait, balance, and mood [53].

Although residents of these communities have lived at high altitude for decades, evidence suggests that compensatory physiological adaptations do not fully restore normal arterial oxygen partial pressure in chronic high-altitude residents [6]. The resulting persistent relative reduction in cerebral oxygen availability may continue to alter serotonin metabolism and sensorimotor function [6,12], possibly contributing to the elevated prevalence of both depressive symptoms and balance disorders observed in our sample. Future studies incorporating biomarkers of altitude adaptation are needed to confirm these mechanisms in chronically adapted Andean populations.

### 4.4. Relevance of the findings

People with depressive symptoms present neurocognitive changes associated with motor coordination [14], with deficits in balance and reduced postural control [11,54,55]. They also show a more stooped appearance, characterized by alterations in body posture and presenting a greater degree of thoracic kyphosis and forward inclination of the head [11]. Stooped posture is also characteristic of aging, which is commonly described as hyperkyphosis and is accompanied by increased postural sway [21]. Postural kyphosis increases the risk of fractures and mortality, and is also associated with deterioration of health, quality of life and physical performance [56,57]. In this sense, it is important to detect depressive symptoms in a timely manner, particularly in older adults, to prevent serious complications associated with balance disorders.

Cultural factors and language barriers can modulate early detection and management of depressive problems in rural populations [58]. The domains and subdomains of intrinsic capacity are interrelated and justify a comprehensive clinical approach to optimize functional ability [59]. The findings of our study demonstrate how we can assess the risk of having balance disorders through the detection of depressive problems. It remains for future research to demonstrate whether the management of depressive problems contributes to improving balance disorders in high Andean populations and vice versa. Given the cross-sectional design of the present study, causality cannot be established; longitudinal studies are needed to confirm the directionality of this association

### 4.5. Limitations and strengths

The present study has some limitations. First, some variables were collected by self-reporting, and therefore, may be affected by recall bias. Nevertheless, 61.6% of the participants did not present cognitive impairment and were interviewed together with a family member to corroborate the information. Future studies should incorporate objective clinical records and validated informant-based instruments to minimize recall bias. Second, the sampling employed was non-probabilistic; however, we included more than 95% of the total potentially eligible older adults in the 12 high Andean communities. Third, the cross-sectional design of the study did not allow the estimation of causal associations between the two main variables. However, our findings provided an important approximation of the association in older adults living at high altitudes to subsequently design longitudinal studies. Longitudinal cohort studies are needed to establish temporal relationships and assess the directionality of this association. Fourth, the GDS-5 and the FRT were administered in Spanish, which may have introduced misclassification in participants whose primary language is Quechua, as cultural and linguistic factors can influence the interpretation of questionnaire items and the execution of physical performance tests. Future studies should incorporate culturally and linguistically adapted versions of these instruments. Additionally, the FRT cut-off of 20.32 cm was originally derived from sea-level populations; its applicability to high-Andean communities warrants further validation. Fifth, altitude of residence varied across communities and, while included as a descriptive variable, was not incorporated as a confounder in the regression models, as it operates at the community level rather than as an individual-level confounder. Future studies with a larger number of communities should consider multilevel modeling approaches that explicitly partition individual- and community-level variance. Sixth, as a secondary analysis, direct measurement of altitude adaptation biomarkers, such as hemoglobin concentration, arterial oxygen saturation, or serotonin metabolism markers, was not feasible. Future studies should incorporate objective physiological measurements to confirm whether hypoxia-related mechanisms are active in chronically adapted Andean populations. The strengths of our study lie in the high initial participation rate and the use of multiple instruments for screening and assessment of geriatric syndromes in older adults in the high Andes.

### 4.6. Conclusions

Depressive symptoms were associated with a higher prevalence of balance disorders in older adults residing in 12 high Andean communities in Peru. Approximately half of the older adults studied presented depressive symptoms or balance disorders, underscoring the significant burden of these conditions in this underserved population. To our knowledge, this is the first study to evaluate this association in rural high-Andean communities, filling an important gap in the geriatric literature for high-altitude settings. These findings have practical implications for primary care: the GDS-5 and the Functional Reach Test are brief, low-cost, validated tools that can be feasibly administered in settings with limited access to health services, and their combined use may help identify older adults at risk for balance disorders and falls. Future epidemiological studies with larger probabilistic samples and longitudinal designs are needed to confirm the directionality of this association and assess the effectiveness of early screening programs in improving functional outcomes and quality of life in older adults living at high altitude.
